# Insight into the CBL and CIPK gene families in pecan (*Carya illinoinensis*): identification, evolution and expression patterns in drought response

**DOI:** 10.1186/s12870-022-03601-0

**Published:** 2022-04-28

**Authors:** Kaikai Zhu, Pinghua Fan, Hui Liu, Pengpeng Tan, Wenjuan Ma, Zhenghai Mo, Juan Zhao, Guolin Chu, Fangren Peng

**Affiliations:** 1 Co-Innovation Center for Sustainable Forestry in Southern China, Nanjing Forestry University, Nanjing, 210037 Jiangsu China; 2 State Key Laboratory of Crop Genetics and Germplasm Enhancement, Ministry of Agriculture and Rural Affairs Key Laboratory of Biology and Germplasm Enhancement of Horticultural Crops in East China, College of Horticulture, Nanjing Agricultural University, Nanjing, 210095 Jiangsu China; 3 Institute of Botany, Jiangsu Province and Chinese Academy of Sciences, Nanjing, 210014 Jiangsu China

**Keywords:** *Carya illinoinensis*, CBL-CIPK, Drought, Evolution, Gene expression

## Abstract

**Background:**

Calcium (Ca^2+^) serves as a ubiquitous second messenger and plays a pivotal role in signal transduction. Calcineurin B-like proteins (CBLs) are plant-specific Ca^2+^ sensors that interact with CBL-interacting protein kinases (CIPKs) to transmit Ca^2+^ signals. CBL-CIPK complexes have been reported to play pivotal roles in plant development and response to drought stress; however, limited information is available about the *CBL* and *CIPK* genes in pecan, an important nut crop.

**Results:**

In the present study, a total of 9 *CBL* and 30 *CIPK* genes were identified from the pecan genome and divided into four and five clades based on phylogeny, respectively. Gene structure and distribution of conserved sequence motif analysis suggested that family members in the same clade commonly exhibited similar exon-intron structures and motif compositions. The segmental duplication events contributed largely to the expansion of pecan CBL and CIPK gene families, and *Ka*/*Ks* values revealed that all of them experienced strong negative selection. Phylogenetic analysis of CIPK proteins from 14 plant species revealed that CIPKs in the intron-poor clade originated in seed plants. Tissue-specific expression profiles of *CiCBLs* and *CiCIPKs* were analysed, presenting functional diversity. Expression profiles derived from RNA-Seq revealed distinct expression patterns of *CiCBLs* and *CiCIPKs* under drought treatment in pecan. Moreover, coexpression network analysis helped to elucidate the relationships between these genes and identify potential candidates for the regulation of drought response, which were verified by qRT–PCR analysis.

**Conclusions:**

The characterization and analysis of *CBL* and *CIPK* genes in pecan genome could provide a basis for further functional analysis of *CiCBLs* and *CiCIPKs* in the drought stress response of pecan.

**Supplementary Information:**

The online version contains supplementary material available at 10.1186/s12870-022-03601-0.

## Background

Calcium ions (Ca^2+^) serve as a secondary messenger that is involved in various signal transduction processes, including growth, development, and response to environmental stimuli in plants [1]. Ca^2+^ signals are commonly perceived by Ca^2+^ sensors, and four Ca^2+^ sensor families have been identified in high plants, such as calcineurin B-like proteins (CBLs), calmodulins (CaMs), calmodulin-like proteins (CMLs), and calcium-dependent protein kinases (CDPKs) [2]. Unlike CDPKs, the CBL, CaM, and CML proteins do not contain any enzymatic domain but transmit Ca^2+^ signals to their downstream targets via their interactors [3].

The CBL protein generally contains an important structural component consisting of four elongation factor hand (EF-hand) motifs for calcium binding. CBL proteins function in various biological processes by activating their downstream targets, CBL-interacting protein kinases (CIPKs), also called (SNF)-related kinases (SnRK3s) [4]. CBL and CIPK form the CBL-CIPK complex to function in different signalling pathways in plants [5]. CIPK proteins are composed of a serine/threonine kinase domain at the N-terminus and a NAF/FISL domain at the C-terminus [6]. The autoinhibitory NAF domain is conserved with 24 amino acid residues, and the activation of plant CIPKs is mediated via the interaction of this domain with CBL proteins [7].

The CBL and CIPK families are two plant-specific gene families, and family members have been identified in numerous species. For example, 10 CBL and 26 CIPK family members were identified in *Arabidopsis*, and 10 CBL and 33 CIPK members were found in rice [1, 8]. Ten CBL and 27 CIPK family members were found in *Populus trichocarpa*, a model woody plant [9, 10]. This phenomenon reveals that CBL may interact with one or more CIPK proteins [11].

Drought is one of the major abiotic stresses that affect plant growth, development, and productivity [12]. The increase in the frequency of drought, with the decrease in soil moisture, is one of the future challenges that affect our society [13]. Understanding the mechanism of how plants respond to drought has been reported at different levels, including epigenetic regulation, metabolic changes, and molecular mechanisms [14]. Numerous genes were upregulated or downregulated under drought, and some of them have been functionally analysed in model plants, such as *Arabidopsis* and rice [15]. The CBL-CIPK signalling pathways were reported to play important roles in responding to various environmental stresses, including drought stress. Multiple *CBL* and *CIPK* genes could be induced under drought stress in grapevine and tea plants [16, 17]. *AtCIPK1* participates in the defence response to drought stress by interacting with both *AtCBL1* and *AtCBL9*, and *cbl1* and *cipk1* mutant plants are hypersensitive to drought stress [18].

Pecan (*Carya illinoinensis* [Wangenh.] K. Koch) is an economically important nut tree of the *Carya* genus that is native to the United States and Mexico, and is now cultivated on six continents [19]. Pecan nuts, the seeds of *C. illinoinensis*, are a rich source of unsaturated fatty acids, vitamins, and numerous bioactive constituents and are commonly consumed snack [20]. The production of pecan nuts was approximately 300 million pounds in the United States in 2020, with a value of approximately $400 million (https://www.nass.usda.gov/). Pecan nut productivity is commonly affected by various biotic and abiotic stresses. Recently, the availability of chromosome-level genome assembly of pecan has allowed us to characterize the pecan CBL and CIPK gene families, and transcriptome data have helped to analyse their expression patterns in different tissues and under drought stress [21]. In the current work, nine *CBL* and 30 *CIPK* genes in pecan were identified, and the evolutionary relationships and duplication events were also analysed. The expression patterns of the two gene family members in various tissues and in response to drought stress were investigated. The precise annotation of the two gene families is the first step to fully understand the roles in pecan drought response.

## Materials and methods

### Identification of the CBL and CIPK gene families in pecan

To identify the CBL and CIPK proteins, all pecan (*Carya illinoinensis*) cv. Pawnee protein sequences were downloaded from the Phytozome database v13 (https://phytozome-next.jgi.doe.gov/) [22]. Ten AtCBL and 26 AtCIPK protein sequences from *Arabidopsis* were retrieved from The Arabidopsis Information Resource (TAIR) database (https://www.arabidopsis.org/). The full-length AtCBL and AtCIPK sequences were aligned with MEGA 7 software (https://www.megasoftware.net) [23]. Then, the alignments were selected to build hidden Markov model (HMM) profiles using the hmmbuild program in HMMER v3.3.2 (http://www.hmmer.org/), and we searched the two HMM profiles against the pecan genome using HMMER with an E-value <1e-10 [24]. The candidate proteins were further examined using Pfam (http://pfam.xfam.org/) and SMART software (http://smart.embl-heidelberg.de/) to confirm the presence of key domains [25]. The EF-hand motif was used for verification of CiCBL family members, while both the kinase and NAF domains were selected for verification of CiCIPK proteins.

The molecular weights (MWs) and isoelectric points (pIs) of CiCBL and CiCIPK proteins were calculated using the ExPASY program (https://web.expasy.org).

### Multiple sequence alignment and phylogenetic analysis

Protein sequences of *Botryococcus braunii* were collected from *Botryococcus braunii* Showa v2.1 DOE-JGI (http://phytozome.jgi.doe.gov/) [26]. Sequences of *Marchantia polymorpha*, *Physcomitrium patens*, *Selaginella moellendorffii*, *Amborella trichopoda*, *Oryza sativa*, *Ananas comosus*, *Betula platyphylla*, *Populus trichocarpa*, and *Salix purpurea* were downloaded from Phytozome 13, common walnut (*Juglans regia*) protein sequences were downloaded from the Genomic Data of Juglans (http://xhhuanglab.cn/data/juglans.html), and Chinese hickory (*Carya cathayensis*) proteins were retrieved from the GigaScience database [27]. The protein sequences of all identified CBL and CIPKs from pecan and the other 13 plant species were used for multiple sequence alignment by MAFFT version 7 (https://mafft.cbrc.jp/alignment/software/) with the G-INS-I program [28]. Phylogenetic trees were then constructed using FastTree software (http://www.microbesonline.org/fasttree/) with the maximum likelihood model [29].

Conserved CBL protein sequences were viewed and edited using GeneDoc software (http://nrbsc.org/gfx/genedoc/). Weblogo (https://weblogo.berkeley.edu/logo.cgi) was then applied to show the features of the NAF domains of CIPK proteins [30].

### Analysis of gene structure and conserved motifs

The coding sequences and genomic DNA sequences of *CiCBL* and *CiCIPK* genes were collected to analyse structural features using TBtools v1.09832 [31].

The amino acid sequences of CiCBLs and CiCIPKs were used to predict the conserved motifs with the MEME online tool (http://meme-suite.org/tools/meme) [32].

### Chromosomal locations of *CBL* and *CIPK* genes in pecan

The chromosomal locations of *CiCBL* and *CiCIPK* genes were retrieved from the phytozome database, and the chromosomal images were visualized using the Circos program in Tbtools software [31].

### Gene duplication and selection pressure analysis

All of the CBL and CIPK protein sequences from the pecan genome were searched against themselves using NCBI-BLAST 2.7.1+ [33]. Collinearity analyses of the CBL and CIPK gene families were performed using MCScanX (Multiple Collinearity Scan toolkit) software (http://chibba.pgml.uga.edu/mcscan2/) [34].

To detect the selection pressure of the duplication events, the CDSs of the *CiCBL* and *CiCIPK* genes were aligned using ClustalW v2.0 software [35]. Then, the synonymous substitution (*Ks*) and nonsynonymous substitution (*Ka*) of tandem and segmental duplication events were calculated using TBtools software, and the selection pressure was evaluated by the *Ka*/*Ks* ratios.

### Plant materials, growth conditions and sample collection

Leaf, mature pistillate and staminate flower, young fruit, and seed samples were collected from nine randomly selected nine-year-old healthy pecan trees of the ‘Pawnee’ cultivar at the experimental station of Nanjing Forestry University, which located in Jurong City, China (119°9′6″E, 31°52′45″N). Ten-month-old seedlings propagated by pecan seeds (collected from ‘Pawnee’ trees) were served as rootstocks. Mature staminate flowers were collected in April, leaves and mature pistillate flowers were harvested in May, young fruits were sampled at 60 days after flowering in July, and seeds were collected in October 2019. Root samples were collected from three-month-old pecan seedlings that were propagated by seeds, and harvested from ‘Pawnee’ trees. All the samples were harvested on sunny days (8:00 to 10:00 am).

For drought treatment, ‘Pawnee’ grafted seedlings were used in this study. The commercial pecan cultivar ‘Pawnee’ was selected as scion, and patch budding was applied for grafting at the experimental station of Nanjing Forestry University in August 2019. Pecan seedlings that propagated by seeds (harvested from ‘Pawnee’ trees in October 2018) were used as rootstocks. The grafted plants were placed in 12-L plastic containers containing a soil mixture of peat, vermiculite, and perlite (5: 3: 2 by volume). After 12 months, the grafted pecan seedlings were then moved to a climate chamber with a photoperiod of 14 h of light at 24 °C/ 10 h of dark at 22 °C, and 60–70% relative humidity. Distilled water was irrigated twice every week. The seedlings were grown in well-watered conditions for 1 month, then they were moved to a growth chamber at 24/22 °C day/night temperatures with a 14/10-h photoperiod, and water was withheld for 15 days. Pecan leaves were collected at 0, 3, 6, 9, 12, and 15 d during drought treatment.

The harvested tissue samples were frozen in liquid nitrogen and stored at − 70 °C until RNA was isolated. Each sample was collected from at least three plants, and three biological repetitions were carried out for each treatment.

### Measurement of proline content and SOD activity

The free proline content was detected according to the ninhydrin method, and the absorbance was measured at 520 nm [36]. Superoxide dismutase (SOD) activity was measured using a Total SOD Assay Kit (A001–1, Nanjing Jiancheng Bioengineering Institute, Nanjing, China) following the manufacturer^’^s instructions.

### RNA isolation and qRT–PCR analyses

Harvested tissue samples were ground to powder in liquid nitrogen. Total RNA was extracted from various tissues using TRIzol reagent (Invitrogen, Carlsbad, USA) following the manufacturer’s instructions. Genomic DNA was removed using a DNase I kit (Qiagen, Hilden, Germany), and RNA quality was detected with an Agilent 2100 bioanalyzer (Agilent Technologies, CA, USA) and quantified using a NanoDrop 2000 spectrophotometer (Thermo Fisher Scientific, Wilmington, USA).

For qRT–PCR (quantitative real-time PCR) analysis, 1 μg of total RNA was used to synthesize first-strand cDNA using the Prime-Script RT Reagent Kit (Takara, Dalian, China). qRT–PCR was performed on an ABI 7500 Real Time PCR system (Applied Biosystems™, Foster City, USA) using TB Green™ Premix Ex Taq™ II (TaKaRa, Shiga, Japan). Specific primers for the *CBL* and *CIPK* genes were designed using the IDT PrimerQuest online tool (https://sg.idtdna.com/PrimerQuest/Home/Index). An actin gene (*CiPaw.03G124400*) that used in previous studies was applied as an internal control for normalization [37]. Relative quantification of *CBL* and *CIPK* genes was determined by the 2^-∆∆Ct^ method [38]. The PCR cycling conditions were as follows: initial denaturation at 95 °C for 30 s, then 40 cycles of 95 °C for 5 s and 60 °C for 15 s.

### Transcriptome analysis

cDNA libraries were constructed and sequenced using an Illumina NovaSeq 6000 platform by Gene Denovo Biotechnology Co. (Guangzhou, China). The FPKM (fragments per kilobase per million of reads mapped) values of *CBL* and *CIPK* genes were calculated by RSEM software to quantify gene expression levels [39]. Sequence data have been uploaded to the NCBI (National Center for Biotechnology Information) database (https://www.ncbi.nlm.nih.gov/) under the accession number GSE179336 and PRJNA799663.

### Coexpression analysis

The Pearson correlation coefficient (PCC) values between different gene pairs of the two family members were calculated with SPSS Statistics 24 based on the expression data of *CiCBLs* and *CiCIPKs* during pecan response to drought treatment. A coexpression network was then built to investigate the relationship between *CiCBL* and *CiCIPK* genes based on PCCs, and the network was then visualized with Cytoscape version 3.8.2 software [40].

### Subcellular localization assays

To investigate the localization of selected *CiCIPKs*, the coding regions of *CiPaw.01G129000*, *CiPaw.07G161900* and *CiPaw.13G065400* were amplified using *Pfu* DNA polymerase (TransGen, Beijing, China), and the PCR products were cloned upstream of a GFP (green fluorescent protein) gene into the pBWA(V)HS vector driven by the 35S promoter (BioRun, Wuhan, China) to generate the constructs. The protoplasts were extracted from 3-week-old *Arabidopsis* seedlings and transformed according to the polyethylene glycol (PEG) method [41]. The transformed protoplasts were incubated at 24 °C for 15–18 h, and then the fluorescence signal was determined under a Nikon C2-ER confocal scanning microscope (Nikon, Kyoto, Japan). Each construct was tested and imaged in at least four protoplasts.

### Statistical analysis

Statistical analyses were carried out using SPSS 24 software. The results were shown as the means ± SE (standard errors) of three biological replicates. One-way ANOVA and Duncan’s multiple range test were selected to compare the significance of differences (*P* < 0.05).

## Results

### Genome-wide investigation of *CiCBL* and *CiCIPK* genes

After screening the pecan genome by domain confirmation, a total of 9 CBLs and 30 CIPKs were identified (Additional file 1: Table S1). Sequence analyses of the two gene family members showed that the full-length *CiCBL* genes varied from 2129 to 13,131 base pairs (bp), and *CiCIPK* genes varied from 1296 to 24,147 bp. The CiCBL and CiCIPK proteins consisted of 179–252/398–492 amino acids (aa), with the relative molecular weights (MWs) of CBLs ranging from 21,121.18 to 29,077.27 Da (Da), while the MWs of CIPKs ranged from 45,423.42 to 55,346.56 Da. Interestingly, the isoelectric points (pIs) of CBL proteins were conserved in pecan, which varied from 4.6 to 4.88, while the pIs of CIPKs varied from 5.67 to 9.39, and 86.67% (26/30) of them had high pIs (pI > 7). All 9 CBL and 30 CIPK proteins were hydrophilic with negative grand average of hydropathicity (GRAVY) values. Detailed information for the two gene family members is listed (Additional file 1: Table S1).

### Gene structural and conserved domain analysis of the pecan CBL and CIPK families

**Fig. 1:**
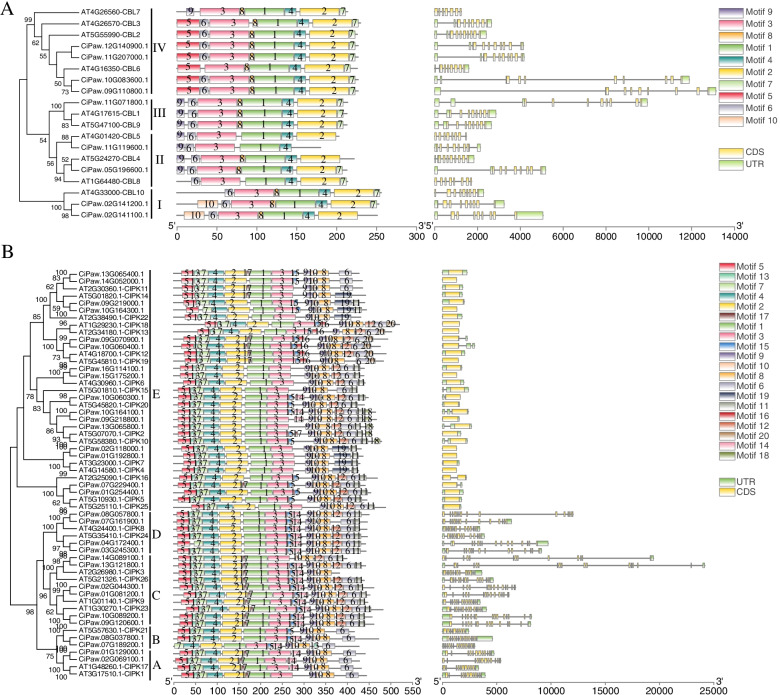
Phylogenetic relationship, gene structure and motif organization analyses of CBLs (**A**) and CIPKs (**B**) from pecan and *Arabidopsis*. The phylogenetic trees on the left included 19 CBL and 56 CIPK proteins from pecan and *Arabidopsis* and are divided into different clades. The conserved motifs are represented by different coloured boxes. Exon/intron structures of *CBL* and *CIPK* genes from pecan are also shown. Green boxes: untranslated regions; yellow boxes: exon regions; black lines: introns

The evolutionary relationships of pecan CBLs and CIPKs were investigated according to phylogenetic trees, which were built using pecan and *Arabidopsis* protein sequences. Nine CiCBLs were divided into four clades (I–V), and Clade IV was composed of the maximum number of 4 members. Thirty CiCIPK proteins were also divided into five clades (A–E), and Clade E contained 16 CIPKs (Fig. 1). The classification result was consistent with previous reports in *Arabidopsis* [17, 42].

Furthermore, the conserved motifs of CBL and CIPK proteins from pecan and *Arabidopsis* were visualized (Fig. 1). Ten different motifs with variable lengths were identified in CBLs from pecan and *Arabidopsis*, and all these proteins contained motifs 1, 3, and 4. Most genes in the same clade exhibited similar motif patterns; for example, CiPaw.02G141100 and CiPaw.02G141200 in clade IV possessed motif 10. Four CiCBLs in clade IV possessed motif 5 (Fig. 1A). The detailed sequence information of the 10 motifs is provided in Additional file 1: Table S2. Twenty conserved motifs were identified in CIPK proteins, and a similar phenomenon also occurred in the pecan CIPK gene family (Fig. 1B). Motifs 5, 13, 7, 4, 2, 1, and 3 at the N-terminus were composed of the catalytic kinase domain. Additionally, motif 9 was the NAF motif, which played a key role in interacting with CBL proteins (Additional file 1: Table S3) [4].

The gene structure analyses showed that each *CiCBL* gene contained multiple introns, which ranged from 7 to 9, while the intron numbers of *AtCBLs* ranged from 6 to 9, suggesting that the intron numbers were relatively conserved (Fig. 1A). However, we found that pecan *CIPK* genes could be divided into two main clusters: an intron-poor cluster (< 4 introns per gene) and an intron-rich cluster (> 10 introns per gene) [43, 44]. All the intron-poor *CiCIPK* genes that contained 0–2 introns were clustered to clade E; however, the intron-rich members containing 11–16 introns were gathered to the other four clades (A–D) (Fig. 1B).

**Fig. 2:**
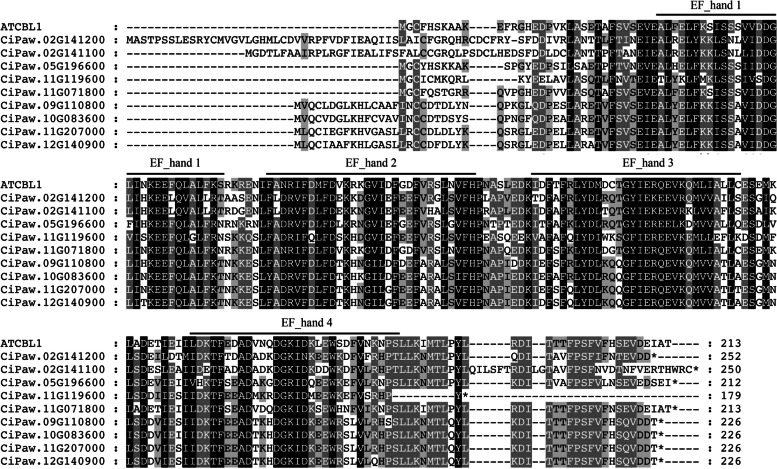
Domain analysis of pecan CBL proteins. Multiple sequence alignment was performed by ClustalW and illustrated by GeneDoc. The four EF-hand motifs were marked by overbars

CBLs are able to bind Ca^2+^ by EF-hands. After comparing the EF-hand motifs of AtCBL1, we also found that four EF-hand motifs existed in all 9 pecan CBL proteins (Fig. 2). Two CiCBLs (CiPaw.05G196600 and CiPaw.11G071800) contained a conserved N-terminal myristoylation site (MGCXXS/T), which might play roles in protein stability and aggregation [45]. CIPK proteins contain an N-terminal protein kinase domain and a NAF domain at the C-terminus. The CBL-CIPK signalling network is mediated by the conserved NAF domain, and our results revealed that the NAF domains in CIPK proteins were highly conserved with many identical residues between *Arabidopsis* and pecan (Additional file 2: Fig. S1).

### Chromosomal location and duplication events among pecan CBL and CIPK genes

**Fig. 3:**
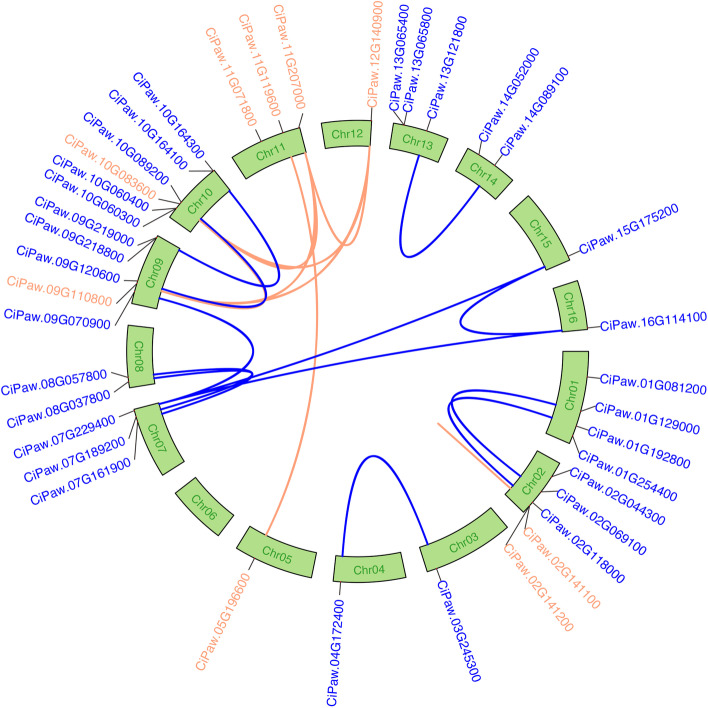
Circos figure for chromosomal locations and interchromosomal relationships of *CBL* and *CIPK* genes from pecan. The *CIPK* genes are marked in blue, and the *CBL* genes are marked in orange. The blue lines in the background represent the gene pairs in the CIPK family, and the orange lines indicate the gene pairs in the CBL family

Genome chromosomal location analyses revealed that 9 *CiCBLs* were distributed on six out of the sixteen chromosomes. Chromosome 11 contained the largest number of *CiCBL* genes, with three, chromosome 2 had two, and the other four were located on chromosomes 5, 9, 10, and 12 (Fig. 3). The 30 *CiCIPK* genes were unevenly mapped on 12 pecan chromosomes, except chromosomes 5, 6, 11, and 12. Specifically, chromosome 10 contained the maximum of 5 *CiCIPK* genes, followed by chromosomes 1 and 9, which both contained 4. In contrast, chromosomes 3, 4, 15 and 16 had only one *CiCIPK* gene (Fig. 3). These results suggested that genetic variations occurred during the evolutionary process of pecan.

Furthermore, MCScanX was applied to analyse the duplication events in the pecan CBL and CIPK gene families. In the pecan CBL gene family, seven segmental duplication events with 8 genes and one tandem duplication event were found, suggesting that most *CiCBL* genes were generated from segmental duplication (Additional file 1: Table S4). In addition, twelve segmental duplication events with 20 *CiCIPKs* were identified (Additional file 1: Table S4). The above results showed that segmental duplication events played a central role in the evolution of *CiCBL* and *CiCIPK* genes.

The synonymous (*Ks*) and nonsynonymous (*Ka*) duplication events were further calculated, and the ratio of *Ka*/*Ks* could be used to explore the selection pressures influencing sequence divergence (Additional file 1: Table S4). A value of *Ka*/*Ks* < 1 indicates negative selection, *Ka*/*Ks* > 1 indicates positive selection, and *Ka*/*Ks* = 1 indicates neutral selection. Amino acid replacements that increased fitness were retained by positive selection, whereas replacements that reduced fitness were removed by negative selection [37]. The *Ka*/*Ks* values of the segmental and tandem duplication events from pecan CBL and CIPK gene families ranged from 0.05 to 0.45, showing that these genes might experience strong negative selection (Additional file 1: Table S4).

### Comparative phylogenetic analyses of the CBL and CIPK families in different species

**Fig. 4:**
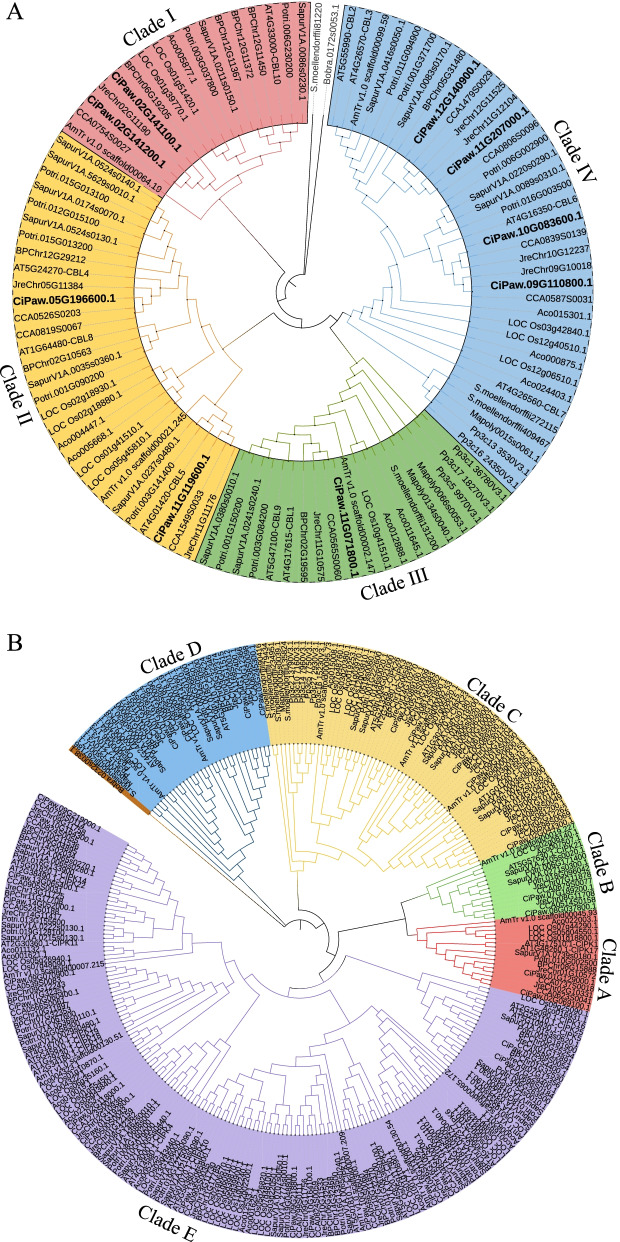
Phylogenetic relationships of the CBL (**A**) and CIPK (**B**) proteins of pecan with those of 13 plant species. The full-length CBL and CIPK protein sequences from *C. illinoinensis*, *C. cathayensis*, *J. regia*, *A. thaliana*, *B. braunii*, *M. polymorpha*, *P. patens*, *S. moellendorffii*, *A. trichopoda*, *O. sativa*, *A. comosus*, *B. platyphylla*, *P. trichocarpa*, and *S. purpurea* were used to build the phylogenetic tree with FastTree. The algae CBL and CIPK proteins were applied as outgroups. The clades are highlighted in different colours

To elucidate the evolutionary relationships among the CBL and CIPK gene families in green algae and land plants, we retrieved candidates of family members from 14 plant genomes, including green algae (*B. braunii*), mosses (*P. patens*), liverworts (*M. polymorpha*), lycophytes (*S. moellendorffii*), basal angiosperms (*A. trichopoda*), monocots (*O. sativa*, *A. comosus*), and eudicots (*A. thaliana*, *B. platyphylla*, *C. illinoinensis*, *C. cathayensis*, *J. regia*, *P. trichocarpa*, and *S. purpurea*). In total, 106 CBL and 283 CIPK candidate proteins were identified from the 14 genomes and applied to construct phylogenetic trees of the CBL and CIPK families (Fig. 4). The numbers of CBLs in the 14 detected species ranged from 1 (*B. braunii*) to 14 (*S. purpurea*), while the numbers of CIPKs varied from 1 (*B. braunii*) to 35 (*O. sativa*, *S. purpurea*). We found that the number of CBLs was commonly smaller than that of CIPKs among these species, except *M. polymorpha*, which contained 3 CBL and 2 CIPK members (Additional file 1: Table S5).

Phylogenetic trees of CBLs and CIPKs were built after sequence alignment analysis. For the phylogeny of the CBL gene family (Fig. 4A), the phylogenetic tree was clearly divided into four clades, which was consistent with our previous results in Fig. 1A. Interestingly, CBL members in Clades I and II were all from seed plants, and CBLs in the moss and liverwort were all grouped in Clades III and IV. For the CIPK gene family (Fig. 4B), the phylogenetic tree was classified into five clades, which was also consistent with previous results (Fig. 1B). The intron-poor CIPK genes were all categorized in Clade E and first appeared in *A. trichopoda*, the basal angiosperm (Fig. 4B). However, *CIPK* genes in the moss, liverwort, and lycophyte all contained multiple introns, which were grouped in Clades C and D.

### Expression profiling of *CiCBL* and *CiCIPK* genes in different pecan tissues

**Fig. 5:**
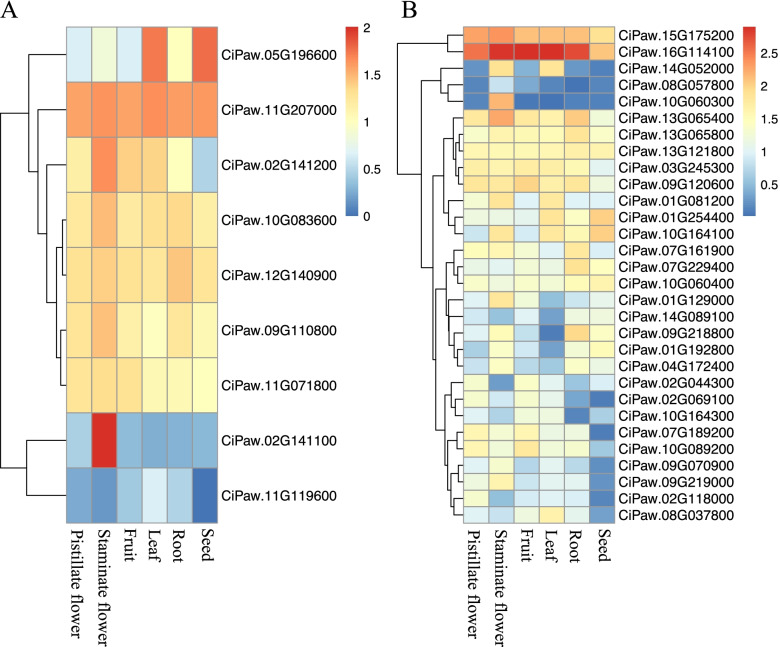
Hierarchical clustering of expression levels of pecan *CBL* and *CIPK* genes in different tissues. **A** Expression patterns of *CBL* genes in various pecan tissues. **B** Expression patterns of *CIPK* genes in various pecan tissues. Heatmaps of *CiCIPK* and *CiCBL* genes were built based on log _10_ (FPKM+ 1) values using R language

The expression profile of a gene might help to elucidate its biological function. To determine the temporal and spatial expression levels of the pecan *CBL* and *CIPK* genes, we investigated the expression profiles of *CiCBLs* and *CiCIPKs* using RNA-Seq data of six different tissues of pecan, including seeds, roots, leaves, young fruits, staminate flowers, and pistillate flowers (Fig. 5). Among the nine *CiCBL* genes, three Clade IV members, *CiPaw.09G110800*, *CiPaw.10G083600* and *CiPaw.12G140900*, exhibited similar expression patterns in different tissues, and the remaining member, *CiPaw.11G207000,* was highly expressed in all six tissues (Fig. 5A). Other *CiCBLs* showed tissue-specific expression patterns. Surprisingly, *CiPaw.02G141100* and *CiPaw.02G141200* in Clade I both showed high expression levels in the staminate flower, indicating that they might function in the development of staminate flowers in pecan. *CiPaw.05G196600* had high expression levels in leaf and seed tissues; however, *CiPaw.11G119600* was expressed at low levels in all detected tissues.

The expression data of thirty *CiCIPK* genes in six tissues showed that different genes exhibited different expression patterns (Fig. 5B). Two CIPK genes (*CiPaw.15G175200* and *CiPaw.16G114100*) generated from segmental duplication both exhibited high expression levels in all tissues. The majority of CIPK genes exhibited tissue-specific expression patterns, suggesting that they play roles in different biological processes. For example, *CiPaw.10G060300* was highly expressed only in staminate flowers, and *CiPaw.08G037800* exhibited high expression levels in leaves. *CiPaw.09G218800*, *CiPaw.01G192800*, and *CiPaw.04G172400* showed relatively high expression levels in the root, seed, and staminate flower tissues.

### Expression patterns and coexpression networks of *CiCBL* and *CiCIPK* genes in response to drought

Drought is one of the most important environmental stress problems that inhibit plant growth [14]. As a positive role in plants response to drought, proline was significantly accumulated in pecan seedlings under drought, especially after treatment for 6 d (Additional file 2: Fig. S2A). The accumulation of reactive oxygen species (ROS) has been found to be stimulated in plants under drought stress, resulting in oxidative stress. Plants were protected against the negative effects of ROS by a complex antioxidant system including SODs, which play a crucial role in the removal of ROS [46]. To investigate the SODs responsible for the scavenging of ROS, the activity of SOD was tested in pecan seedlings after drought treatment, and we found the SOD activity also increased significantly (Additional file 2: Fig. S2B).

**Fig. 6:**
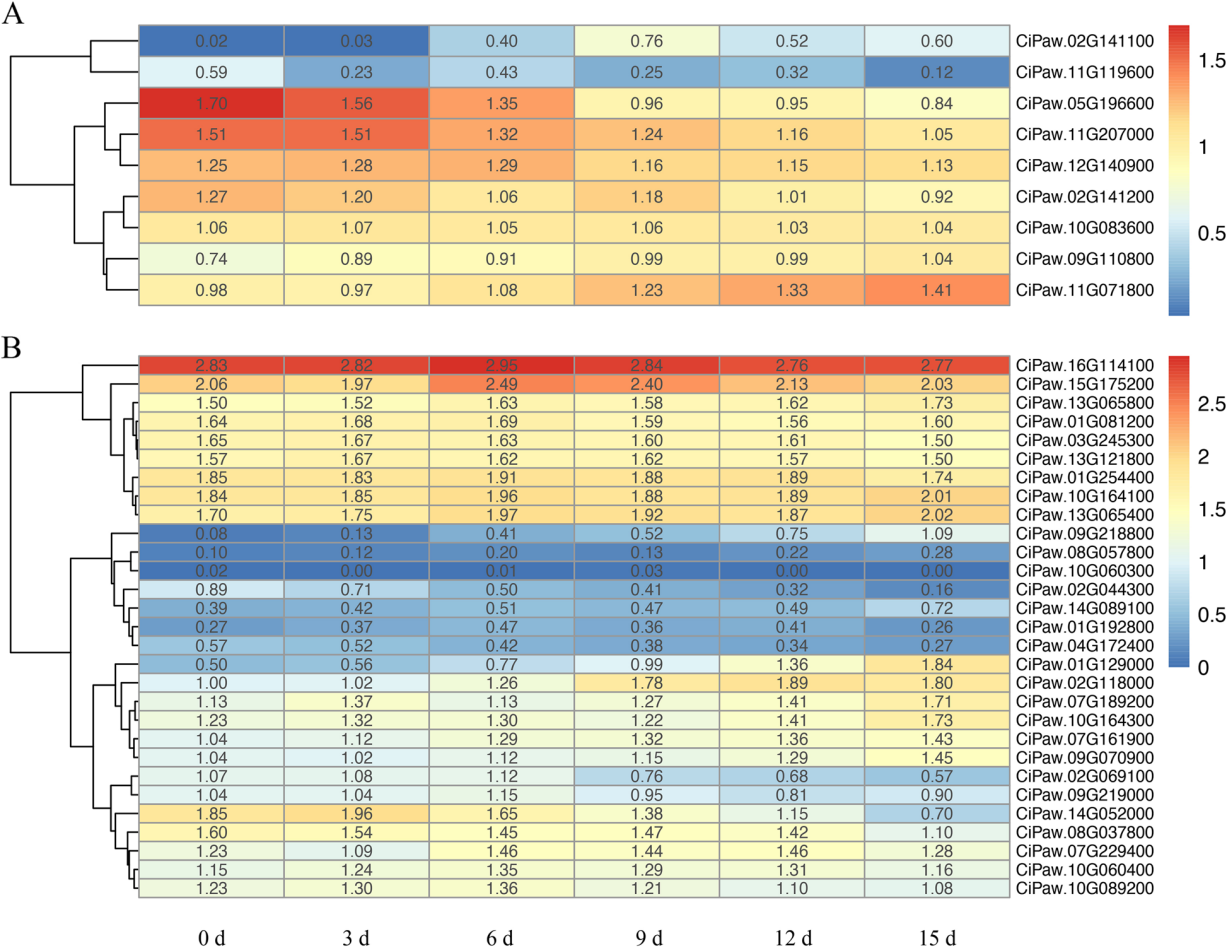
Hierarchical clustering of expression levels of pecan *CBL* and *CIPK* genes in response to drought. **A** Expression patterns of *CBL* genes under drought. **B** Expression patterns of *CIPK* genes under drought. Heatmaps of *CiCIPK* and *CiCBL* genes were built based on log _10_ (FPKM+ 1) values using R language

The CBL-CIPK module has been reported to be involved in the response to environmental stresses, especially drought stress [42, 47]. To investigate the roles of *CiCBLs* and *CiCIPKs* in response to drought, their expression profiles were analysed using RNA-Seq datasets. In total, 9 available *CBL* and 29 *CIPK* genes showed their expression patterns under drought stress (Fig. 6). Two *CiCBL* genes (*CiPaw.02G141100* and *CiPaw.11G119600*) showed low expression levels under drought treatment, and *CiPaw.05G196600* and *CiPaw.11G207000* gradually decreased and showed the lowest expression levels after 15 days of drought application (Fig. 6A). In contrast, *CiPaw.09G110800* and *CiPaw.11G071800* were gradually upregulated by drought at different time points. Twenty-nine *CiCIPK* genes showed various expression patterns in pecan subjected to drought stress (Fig. 6B). Six *CiCIPKs* were expressed at low levels, while three genes were downregulated. Most of the remaining *CIPK* genes were upregulated, ten of which gradually increased and peaked at 15 days of drought application.

A coexpression network was further built to explore the mutual relationships of pecan *CBL* and *CIPK* genes by analysing their transcript levels under drought. Within the network, 34 nodes (9 *CBLs* and 25 *CIPKs*) and 172 edges were found, and 17 nodes contained more than ten edges, suggesting that these nodes were tightly correlated (Additional file 2: Fig. S3). *CiPaw.11G071800* contained the maximum numbers of edges, which included 5 *CBLs* and 15 *CIPKs*. According to the 172 coexpression events, 105 exhibited significantly positive correlations, and the remaining 67 exhibited significantly negative correlations.

**Fig. 7:**
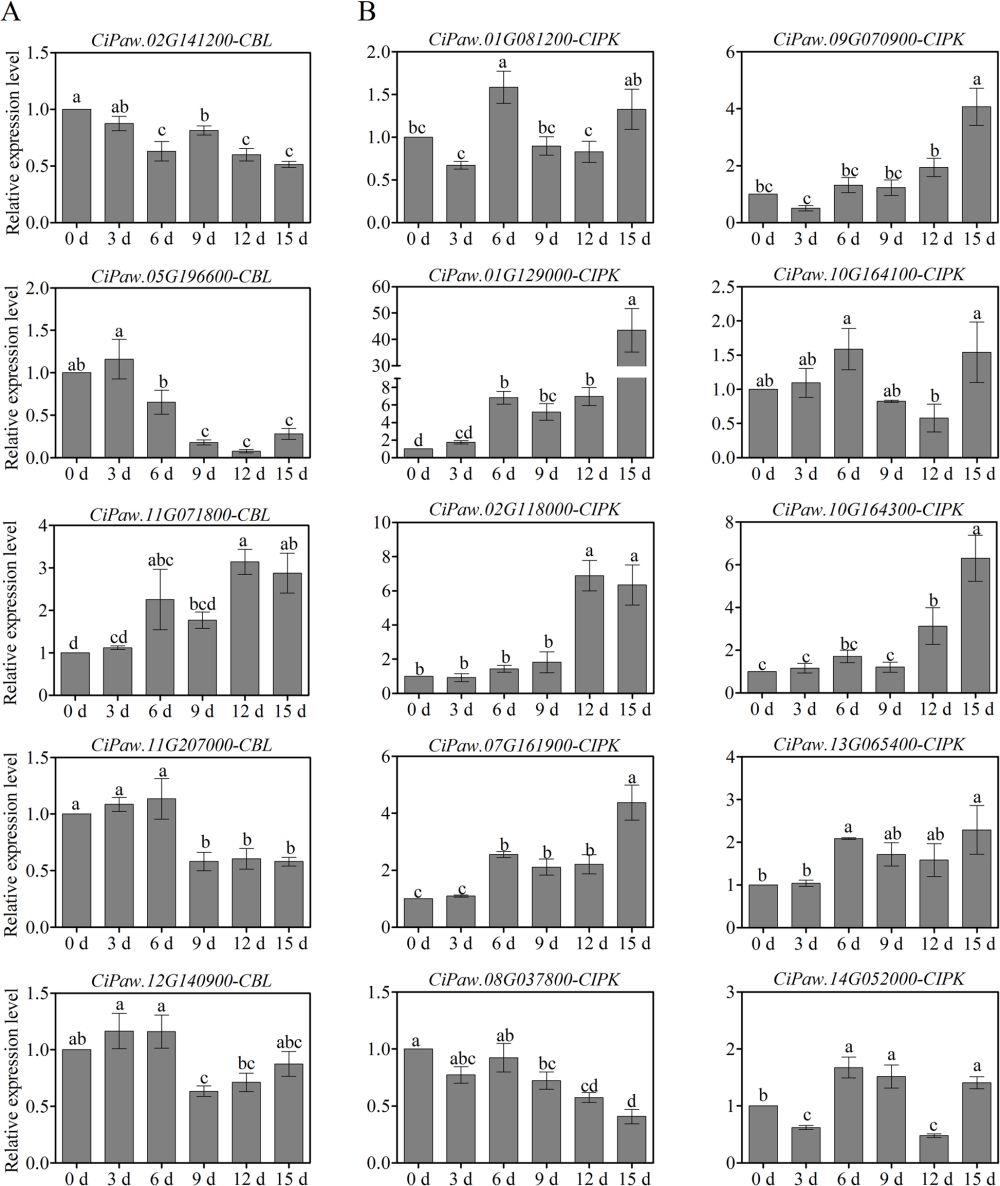
Expression analysis of *CiCBL* and *CiCIPK* genes in pecan exposed to drought stress. Expression patterns of 5 *CiCBL* (**A**) and 10 *CiCIPK* (**B**) genes were analysed using qRT–PCR. The actin gene (*CiPaw.03G124400*) was selected as the reference gene. Lowercase letters represent significant differences (*P* < 0.05) according to Duncan’s multiple range test. Error bars indicate the means ± SE obtained from three biological replicates

To validate the reliability of the RNA-Seq data, qRT–PCR was applied to analyse the expression levels of pecan *CBL* and *CIPK* genes under drought stress. Based on the RNA-Seq and coexpression results, 5 *CiCBL* and 10 *CiCIPK* genes were selected for further confirmation after drought stress was imposed (Fig. 7). The specific primers used in the study were listed in Additional file 1: Table S6. As shown in Fig. 7A, the expression patterns of five *CiCBL* genes were consistent with the previous RNA-Seq results (Fig. 6). Four *CiCBLs* were downregulated, while *CiPaw.11G071800* was significantly induced after 12 and 15 d of drought treatment. The expression patterns of 90% *CiCIPKs* were consistent between RNA-Seq and qRT–PCR results, except for *CiPaw.14G052000*, which exhibited different expression patterns (Fig. 7B). Unlike *CiCBLs*, most *CIPK* genes were enhanced by drought treatment, especially after 15 d of treatment, indicating that they might play roles in the response to drought. For example, *CiPaw.01G129000* was gradually upregulated under drought stress and displayed the highest expression level at 15 days after treatment, which was more than 40-fold higher than that of the control (0 d).

### Subcellular localization of CiCIPKs

**Fig. 8:**
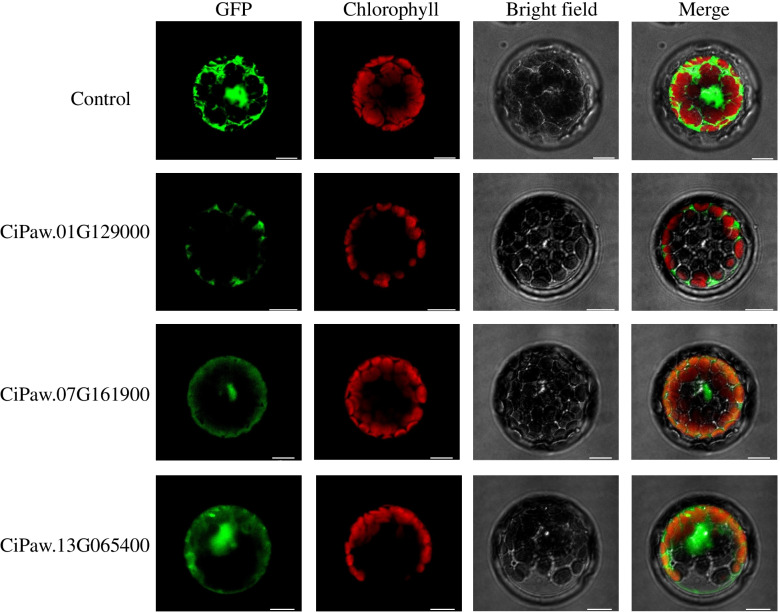
Subcellular localization of three CiCIPKs based on the transient expression of fused GFPs. Control, GFP alone. Bar = 10 μm

The subcellular localization of a protein might help to reveal its function. According to the expression (Figs. 6 and 7) and coexpression analysis (Additional file 2: Fig. S3), three *CiCIPK* genes (*CiPaw.01G129000*, *CiPaw.07G161900*, and *CiPaw.13G065400*) that coexpressed with more than ten genes were induced gradually by drought treatment, and showed the highest expression levels after 15 d of drought. These *CiCIPKs* were selected as candidate genes, and the coding regions of the three genes were fused to the green fluorescent protein (GFP) reporter gene in the binary vectors. Then, the recombinant constructs were transiently expressed in *Arabidopsis* protoplasts. The results revealed that the control GFP was distributed throughout the cell, whereas the CiPaw.01G129000-GFP fluorescence signal was only found in the cytoplasm, suggesting that the CiPaw.01G129000 protein was localized to the cytoplasm. Moreover, the CiPaw.07G161900-GFP and CiPaw.13G065400-GFP fusion proteins were detected in both the cytoplasm and nucleus (Fig. 8).

## Discussion

The role of calcium in stress signal transduction has been well established in plants [48]. CBLs are plant-specific Ca^2+^ sensors that interact with and activate CIPKs to transduce Ca^2+^ signals [4]. To date, CBL and CIPK family members have been identified in model plants and major crops, including *Arabidopsis*, canola, rice, and poplar [8, 9, 49]. However, very little is known in pecan.

In the current study, 9 CiCBL and 30 CiCIPK members were found in the pecan genome (Fig. 1). Similar to those CBL proteins in *Arabidopsis* and canola [49], four EF-hand motifs were found in the nine CiCBL proteins (Fig. 2). As a type of Ca^2+^ sensor, CBL proteins are able to bind Ca^2+^ through EF-hand motifs [10]. In addition, two CiCBL proteins started with a conserved N-terminal myristoylation site, which might play a role in membrane association of the CBL-CIPK complex by switching calcium with myristoyl [50]. CIPK proteins commonly possess an N-terminal kinase domain and a conserved C-terminal NAF domain, and all CiCIPKs contained these two domains (Fig. 1B). CBLs bind to the short NAF domains to release from the kinase domain and switch the kinase into an active conformation [51].

Comparison of the numbers of *CBL* and *CIPK* genes in pecan with other sequenced plant genomes revealed that the two gene families have expanded multiple times in evolutionary history (Additional file 1: Table S5) [52]. In the green algae *Botryococcus braunii*, both CBL and CIPK contained only one family member, and the family sizes increased quickly following evolution to seed plants. Gene duplication commonly plays a key role in the expansion of a gene family during evolution and functions in adaptation to environments by obtaining new gene functions in plants [53]. Segmental/whole-genome duplications and tandem duplications contribute to the evolutionary expansion of gene families [54]. In pecan, segmental duplication occurred in both CiCBLs and CiCIPKs, and contributed largely to the expansion of the two families, while only one tandem duplication event was detected in the CBL family (Fig. 3). CIPKs resulting from both segmental and tandem duplications were found in *Arabidopsis*, rice, maize, and grape [16, 43]. In contrast, only tandem duplication events were detected in grape CBLs, and tandem duplications played major roles in *Medicago* CBL and CIPK families [16, 55]. Negative selection functions in the process of removing deleterious mutations to prevent functional divergence; however, positive selection plays a role in new advantageous mutation collection and spreading throughout the population [56]. The *Ka*/*Ks* ratios of all the segmental and tandem duplication events in pecan CBLs and CIPKs showed that they were driven by negative selection (Additional file 1: Table S4). Gene duplication can lead to accumulate degenerative mutations, and negative selection may result in stabilizing selection via removing deleterious variations that arise, indicating these *CiCBLs* and *CiCIPKs* under negative selection were functionally conserved, respectively [57]. The *CBL* and *CIPK* genes of *Medicago* have also undergone strong negative selection pressure during evolution [55].

According to the phylogenetic analysis, pecan CBLs and CIPKs were classified into four and five clades, which was consistent with previous findings in grape and pepper (Fig. 4) [16, 58]. Gene structures such as exon-intron organizations and intron numbers are imprints of evolution in several gene families [59]. Eukaryotic genes could be divided into intron-rich, intron-poor (less than four introns), or intronless (no introns), and genes of early-diverging plant species were mostly intron-rich [60]. The intron numbers in *CiCBLs* were very conserved, ranging from 7 to 9; however, the *CiCIPKs* were clearly divided into an intron-rich cluster (clades A, B, C, and D) and an intron-poor cluster (clade E) (Fig. 1). This phenomenon of gene structure in *CIPK* genes widely exists in *Arabidopsis*, canola, grape, and soybean [1, 16, 42, 49]. Interestingly, the intron-poor cluster members in the CIPK gene family first appeared in seed plants, while *CIPKs* in algae, moss, spikemoss, and fern all possessed multiple introns (Fig. 4) [5, 42]. These results suggested that intron loss and gain events functioned in the evolution of plant *CIPK* genes. Interestingly, clustering expression results suggested that *Arabidopsis CIPK* genes could be induced by environmental stresses, and some CIPK genes in the two clusters shared similar expression patterns in response to environmental stresses [43]. For example, three *CIPKs* (*CIPK8*, *CIPK21* and *CIPK24*) in intron-rich cluster and two (*CIPK6* and *CIPK13*) in intron-poor cluster were all involved in the regulation of salt stress [61–65]. For CBLs, proteins in the same clade have high sequence similarities (Fig. 2), when fused with GFP, three CBLs including CBL2, CBL3 and CBL6 in clade IV were localized at the tonoplast, while CBL1 and CBL9 in Clade III were both detected at the plasma membrane [66]. Moreover, CBL1 and CBL9 both functioned in K^+^ homeostasis under low K^+^ condition via interacting with CIPK23 in *Arabidopsis* [67]. These two CBLs also play a crucial role in pollen germination and tube growth [68]. CBL2 and CBL3 coexpressed with CIPK21 to respond to salt stress by regulating ion and water homeostasis [63]. Although genes in the same clade were considered to be functionally identical, CIPKs and CBLs with high sequence similarities may show distinct functions, such as CBL1 showed distinctively different functions from CBL9 in response to ABA treatment [69].

Drought is a major environmental stress that profoundly affects the growth, development, and productivity of plants. CBL-CIPK complexes have been previously proven to play important roles in drought stress signalling pathways [42]. Our RNA-Seq data under drought treatment revealed that the expression levels of some *CiCBLs* and *CiCIPKs* were drastically changed (Fig. 6). Most *CiCBL* genes were downregulated in response to drought, except *CiPaw.11G071800*, which was significantly induced (Fig. 7A). Orthologous *AtCBL9* from *Arabidopsis* has been shown to be essential for the drought stress response. *AtCBL9* was involved in the drought-induced abscisic acid (ABA) production process, which was highly inducible by drought and treatments with the plant hormone ABA, and the *cbl9* mutant seedlings were more sensitive to drought [70]. Unlike *CiCBLs*, the majority of *CiCIPK* genes were upregulated under drought stress (Fig. 6B), which was further validated by qRT–PCR (Fig. 7B). As protein kinases, CIPKs play central roles in the response to drought in plants, and RNA-Seq results of drought treatment in grapevine revealed that the expression levels of many kinase genes changed, including *CIPKs* [71]. Interestingly, most *CIPKs* in soybean and cassava were drought-responsive genes, especially family members belonging to the intron-poor clade [42, 72]. *AcCIPK18*, an intronless *CIPK* gene in pineapple, is a positive regulator of drought stress, and the overexpression lines showed significantly stronger drought tolerance than wild-type plants [73]. *CaCIPK3* is an intronless gene in pepper that participates in the response to various stresses, including drought. Knockdown of *CaCIPK3* improves sensitivity to drought, while overexpression of *CaCIPK3* increases drought tolerance by enhancing the activities of antioxidant defence systems [47]. The CcCBL1-CcCIPK14 complex positively regulates drought tolerance through modulation of flavonoid biosynthesis in pigeon pea. CcCIPK14-overexpressing (*CcCIPK14-OE*) plants had enhanced drought tolerance, but this phenomenon was reversed in *CcCBL1*-RNAi *CcCIPK14*-OE double transgenic plants [74].

In conclusion, a total of 9 CBLs and 30 CIPKs were annotated in pecan at the genome scale and were classified into four and five clades, respectively. The gene structure and conserved domains, chromosome locations, duplication events, evolutionary relationships, and expression patterns of *CiCBL* and *CiCIPK* genes were investigated. The results indicated that CiCBLs and CiCIPKs might function in development and drought response in pecan. Overall, this research provides important information concerning pecan CBLs and CIPKs, and will be helpful for further functional investigation.

## Supplementary Information

**Additional file 1: Table S1.** *CBL* and *CIPK* genes identified in pecan and their sequence characteristics. **Table S2.** Motif sequences of pecan CBLs. **Table S3.** Motif sequences of pecan CIPKs. **Table S4.** Duplication events and related *Ka/Ks* values of pecan CBLs and CIPKs. **Table S5.** The CBL and CIPK protein numbers in different plant species. **Table S6.** Specific primers of pecan *CBL* and *CIPK* genes for qRT-PCR analysis.

**Additional file 2: Figure S1.** Highly conserved NAF domain across CIPK proteins in pecan (A) and *Arabidopsis* (B). Multiple alignment analysis of CIPK domains was presented by ClustalW and sequence logos were generated by Weblogo. **Figure S2.** Analysis of proline content (A) and SOD activity (B) of pecan seedlings in response to drought. Lowercase letters represent significant differences (*P* < 0.05) according to Duncan’s multiple range test. Error bars indicate the means ± SE obtained from three biological replicates. **Figure S3.** The coexpression network of *CBL* and *CIPK* genes in response to drought in pecan. The nodes indicate different genes, and the edges between nodes indicate coexpression correlations of gene pairs (*P* < 0.05). Edge line colours indicate either positive (red, PCC ≥ 0.6) or negative (blue, PCC ≤ − 0.6) correlations.

## Data Availability

All the datasets used for this research are available on reasonable request from the corresponding author. The RNA sequencing raw data can be found in the NCBI BioProject (PRJNA799663) and Gene Expression Omnibus (GEO) database (GSE179336), respectively.

## Abbreviations

CBL: Calcineurin B-like protein
CIPK: CBL-interacting protein kinase
EF-hand: Elongation factor hand
FPKM: Fragments per kilobase per million of reads mapped
GFP: Green fluorescent protein
GRAVY: Grand average of hydropathicity
HMM: Hidden Markov model
*Ka*: Nonsynonymous substitution
*Ks*: Synonymous substitution
MW: Molecular weight
PCC: Pearson correlation coefficient
PEG: Polyethylene glycol
pI: Isoelectric point
qRT–PCR: Quantitative real-time PCR
RNA-Seq: RNA sequencing
SOD: superoxide dismutase
