# Supplementary material for: Insight into the CBL and CIPK gene families in pecan (Carya illinoinensis): identification, evolution and expression patterns in drought response
Source: BMC Plant Biol. 2022 Apr 28;22:221. doi: 10.1186/s12870-022-03601-0 (PMC9047272; doi:10.1186/s12870-022-03601-0)
Supplement: Supplementary file 2 — Additional file 2: Figure S1. Highly conserved NAF domain across CIPK proteins in pecan (A) and Arabidopsis (B). Multiple alignment analysis of CIPK domains was presented by ClustalW and sequence logos were generated by Weblogo. Figure S2. Analysis of proline content (A) and SOD activity (B) of pecan seedlings in response to drought. Lowercase letters represent significant differences (P < 0.05) according to Duncan’s multiple range test. Error bars indicate the means ± SE obtained from three biological replicates. Figure S3. The coexpression network of CBL and CIPK genes in response to drought in pecan. The nodes indicate different genes, and the edges between nodes indicate coexpression correlations of gene pairs (P < 0.05). Edge line colours indicate either positive (red, PCC ≥ 0.6) or negative (blue, PCC ≤ − 0.6) correlations. [file 12870_2022_3601_MOESM2_ESM.docx]

**Fig. S1**

**Fig. S1** Highly conserved NAF domain across CIPK proteins in pecan (A) and *Arabidopsis* (B). Multiple alignment analysis of CIPK domains was presented by ClustalW and sequence logos were generated by Weblogo.

**Fig. S2**

**
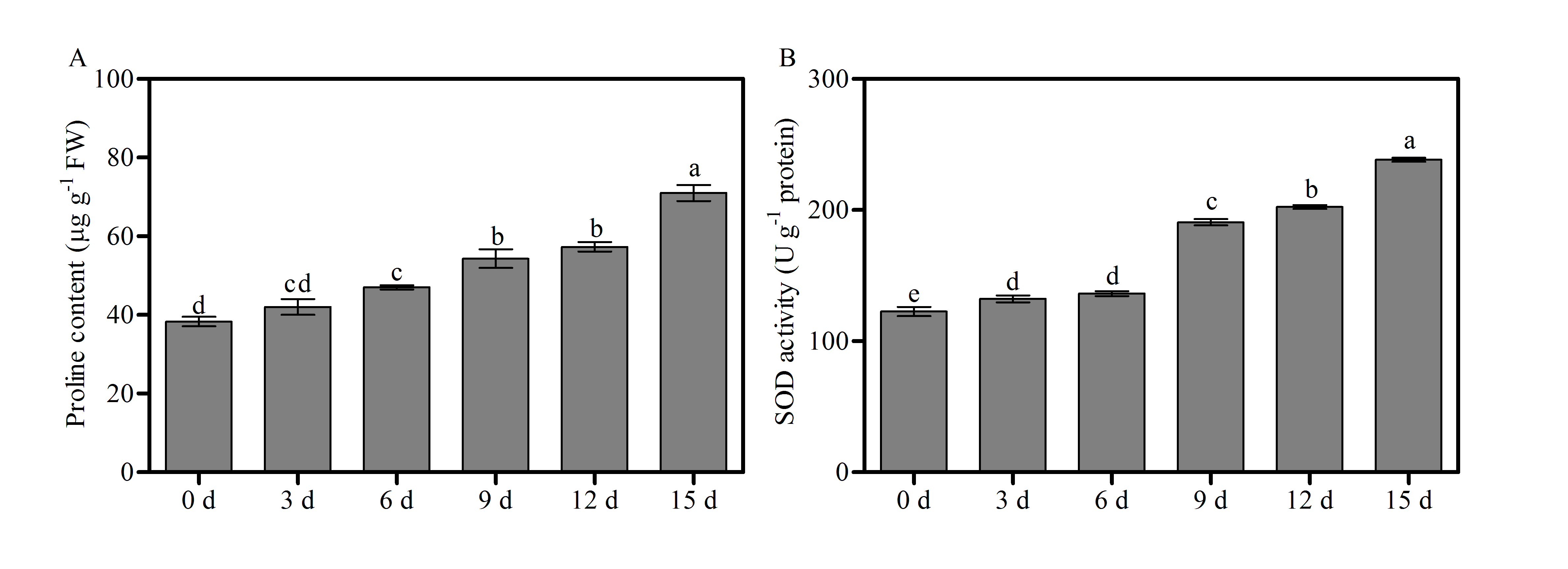
**

**Fig. S2** Analysis of proline content (A) and SOD activity (B) of pecan seedlings in response to drought. Lowercase letters represent significant differences (*P* < 0.05) according to Duncan’s multiple range test. Error bars indicate the means ± SE obtained from three biological replicates.

**Fig. S3**

**Fig. S3** The coexpression network of *CBL* and *CIPK* genes in response to drought in pecan. The nodes indicate different genes, and the edges between nodes indicate coexpression correlations of gene pairs (*P* < 0.05). Edge line colours indicate either positive (red, PCC ≥ 0.6) or negative (blue, PCC ≤ -0.6) correlations.
